# The Book World of Medicine and Science

**Published:** 1897-06-12

**Authors:** 


### THE BOOK WORLD OF MEDICINE AND SCIENCE.

The Treatment of Lupus. By Balmanno Squire, M.B. London, Surgeon to the British Hospital for Diseases of the Skin, King's Cross. (London: J. and A. Churchill, 7, Great Marlborough Street. 1897. Pamphlet form, 62 pages. Frontispiece.)

The author, after enumerating the graver consequences of this disease, calls attention to the fact that its onset is usually in comparatively harmless situations. The skin alone is invaded and deeper structures only implicated when the skin itself is destroyed. Complete extirpation is always difficult, sometimes impossible. Common causes of failure are probab'y due to the idea that the apple-jelly deposit is the only lupus tissue, and that the visible limits of the disease are its real limits; to the more or less unprecise methods of operating in vogue; and to the customary administration of anæsthetics, which by flushing the surface obscure the smaller patches from view. The author's method aims at further precision in operating whether by scraping or puncturation. His usual plan is to outline the patches with a solution of black sealing-wax, including a zone of sound skin. Local anæsthesia is produced by freezing. This, besides preventing bleeding, renders the part firm and tense, so that there is no dragging of the skin under the curette. The scraping process is less a scraping than a "conservative excision," the curette gouging out the frozen patches. The instrument used has a thin handle, and is held like a pencil. The spoon is very small, with a sharp edge. Three or four days after the first operation the wound is again frozen and treated by linear scarification with a specially-devised instrument as an accessory precaution. No other method out of many employed by the author during a number of years and on a large number of cases has given such satisfactory results.

Twentieth Century Practice. Edited by Thomas Stedman, M.D. Vol. VIII. "Diseases of the Digestive Organs." (London: Sampson Low, Marston, and Co. 1897.)

Commencing with diseases of the mouth, this volume deals in succession with diseases of the œsophagus, the stomach, the pancreas, and the peritoneum, ending with a chapter on animal parasites and the diseases caused by them. The chapter on the examination of the stomach is very complete, many methods being described which, up to the present, are far from being in common use. Such are gastro-diaphany, or transillumination of the stomach, by means of an electric light introduced within it; the use of the "stomach bucket," a small silver capsule attached to a silk thread, which is swallowed and then withdrawn, bringing back with it a sample of the stomach contents; and the examination of the muscular activity of the organ by means of the gastrograph. The uncertainty of the deductions from the use of some of these methods is however greatly increased by the difficulty of obtaining control observations on a sufficiently large number of healthy people. The article, taken as a whole, is a very useful *résumé* of what is known, and of a good deal which is hardly yet known, on the very large subject of diseases of the stomach. The article on diseases of the peritoneum is made to include appendicitis, perforation of the stomach and intestine, subphrenic abscess, and peritonitis originating from the female generative organs. It thus becomes to a large extent surgical, and is written by a surgeon, Dr. Farquhar Curtis, of New York. The article on diseases of the pancreas, by Dr. Hans Leo, of Bonn, is a very good one, and gives much information on a subject which is far too sketchily treated in many works on medicine. The last 150 pages of the book are devoted to the animal parasites, of which a very full, complete, and well-illustrated account is given.

Who's Who, 1897. Edited by Douglas Sladen. First year of new issue. (London: A. and C. Black. Price 3s. 6d.)

This is undoubtedly a most useful book to have at hand upon one's desk. The multitude of people who are somebodies is so great, and the difficulty of remembering the grounds on which the individuals in the crowd have become known to fame is one which increases so rapidly year by year, that we anticipate a steadily growing popularity for this book. Some people may doubt the advantage of publishing biographical details so obviously supplied by the people concerned, but we are not sure that we do not learn more of a man's character by reading what he chooses to say about himself than by merely accepting the opinion of any editor, however sharp; and we think the editor of this volume has done well to let his subjects speak for themselves. Although the major part of the book is occupied by biographical notices, it contains a great deal of information which will be found very useful, chiefly in the form of lists of people having titles, or occupying official positions, or being in any way so marked out from their fellows as to be capable of convenient grouping. The work is interesting—sometimes even amusing—and reflects great credit on the editor, who will, we fancy, find his labours grow as years roll by, for the task of selection will become a very difficult one when increasing popularity brings long lists of candidates anxious to see the trivial details of their lives recorded in its pages.

Compressed Air Illness, or So-called Caisson Disease. By E. Hugh Snell, M.D., B.Sc., Lond. 8vo. pp. 251. (London: H. K. Lewis. 1897.)

The recent Royal opening of the latest wonder of engineering skill has drawn very widespread attention to the peculiar circumstances under which a large part of the work had to be carried on. The tunnel is probably the pioneer of several other future engineering feats which will render the workman subject to disease from long hours spent in highly compressed air. Thus, not only is the work before us an interesting and original clinical description of an almost unknown affection, but it gains in value from the fact that the condition is sure to be encountered by future practitioners, who will be very glad to know where to turn for information and advice. Dr. Snell's work is the only essay on the subject in the English language, and for collated observations it is probably the most complete that has yet been published.

### BOOKS RECEIVED.

Bailliere, Tindall, and Cox.

"From Our Dead Selves to Higher Things." By James Gant, F.R.C.S.

"Excretory Irritation." By David Walsh, M.D.

The Cotton Press.

"Molière and his Medical Associations." By A. M. Brown, M.D.

Sampson Low, Marston, and Co.

